# Two Sides of the Same Coin: Protein Kinase C γ in Cancer and Neurodegeneration

**DOI:** 10.3389/fcell.2022.929510

**Published:** 2022-06-21

**Authors:** Caila A. Pilo, Alexandra C. Newton

**Affiliations:** ^1^ Department of Pharmacology, University of California, San Diego, San Diego, CA, United States; ^2^ Biomedical Sciences Graduate Program, University of California, San Diego, San Diego, CA, United States

**Keywords:** protein kinase C, autoinhibition, spinocerebellar ataxia, cancer, neurodegeneration

## Abstract

Protein kinase C (PKC) isozymes transduce myriad signals within the cell in response to the generation of second messengers from membrane phospholipids. The conventional isozyme PKCγ reversibly binds Ca^2+^ and diacylglycerol, which leads to an open, active conformation. PKCγ expression is typically restricted to neurons, but evidence for its expression in certain cancers has emerged. PKC isozymes have been labeled as oncogenes since the discovery that they bind tumor-promoting phorbol esters, however, studies of cancer-associated PKC mutations and clinical trial data showing that PKC inhibitors have worsened patient survival have reframed PKC as a tumor suppressor. Aberrant expression of PKCγ in certain cancers suggests a role outside the brain, although whether PKCγ also acts as a tumor suppressor remains to be established. On the other hand, PKCγ variants associated with spinocerebellar ataxia type 14 (SCA14), a neurodegenerative disorder characterized by Purkinje cell degeneration, enhance basal activity while preventing phorbol ester-mediated degradation. Although the basis for SCA14 Purkinje cell degeneration remains unknown, studies have revealed how altered PKCγ activity rewires cerebellar signaling to drive SCA14. Importantly, enhanced basal activity of SCA14-associated mutants inversely correlates with age of onset, supporting that enhanced PKCγ activity drives SCA14. Thus, PKCγ activity should likely be inhibited in SCA14, whereas restoring PKC activity should be the goal in cancer therapies. This review describes how PKCγ activity can be lost or gained in disease and the overarching need for a PKC structure as a powerful tool to predict the effect of PKCγ mutations in disease.

## Introduction

The protein kinase C (PKC) branch of the AGC kinase family tree is encoded by nine genes to yield 10 isozymes. These share a similar primary sequence and 3D architecture, yet are differentially regulated by second messengers to transduce a diverse range of signals within the cell. The conventional PKC isozymes (α, βI/II, and γ) are the most well-characterized, with signaling of these isozymes being tightly restricted to ensure activation only in response to appropriate stimuli. In the absence of second messengers, these enzymes maintain an autoinhibited state by a set of N-terminal regulatory domains that prevent the C-terminal kinase domain from phosphorylating its substrates (Newton, 2018). Within the active site, an autoinhibitory pseudosubstrate region binds to maintain the enzyme in an inactive conformation. The DG-sensing C1 domains and Ca^2+^-sensing C2 domain pack against the kinase domain to maintain it in an autoinhibited conformation (Antal et al., 2015a). Binding of DG and Ca^2+^ permits pseudosubstrate release from the active site and substrate phosphorylation. These second messengers are generated upon receptor-mediated hydrolysis of phosphatidylinositol 4,5-bisphosphate (PIP_2_) into DG and IP_3_, which causes Ca^2+^ release into the cytosol. Binding of Ca^2+^ to the C2 domain leads to plasma membrane engagement and PIP_2_ binding (Evans et al., 2006). At the plasma membrane, the C1B domain binds DG, and the C1A domain assists in pseudosubstrate release from the active site (Antal et al., 2014). Second messenger metabolism leads to a decrease in PKC activity due to re-autoinhibition.

To gain this autoinhibited state, newly-translated PKC undergoes a series of priming phosphorylations involving mammalian target of rapamycin (mTOR) complex 2 (mTORC2), pyruvate dehydrogenase kinase 1 (PDK-1), and autophosphorylation (Baffi and Newton, 2022). Autophosphorylation at the C-terminal hydrophobic motif is necessary for PKC to adopt the autoinhibited conformation (Baffi et al., 2019). PKC that is not properly autoinhibited is dephosphorylated by the PH domain and leucine rich repeat protein phosphatase (PHLPP), and subsequently shunted to a degradative pathway (Baffi et al., 2019). This PHLPP-mediated dephosphorylation of PKC acts as a quality control mechanism, ensuring that only properly autoinhibited PKC accumulates in the cell. For example, cancer-associated PKC mutants that impair PKC autoinhibition, including cancer fusion proteins, are paradoxically loss-of-function because mutant protein is degraded by this quality control pathway (Baffi et al., 2019; Van et al., 2021). In this way, prolonged activation promotes the dephosphorylation and degradation of PKC. Additionally, tumor-promoting phorbol esters, which lock PKC in an open and active conformation, lead to dephosphorylation and degradation of PKC (Hansra et al., 1999). Thus, phorbol esters lead to acute activation, but ultimately downregulation, of PKC (Jaken et al., 1981).

### PKC Isozymes Share a Common 3D Architecture

**FIGURE 1:**
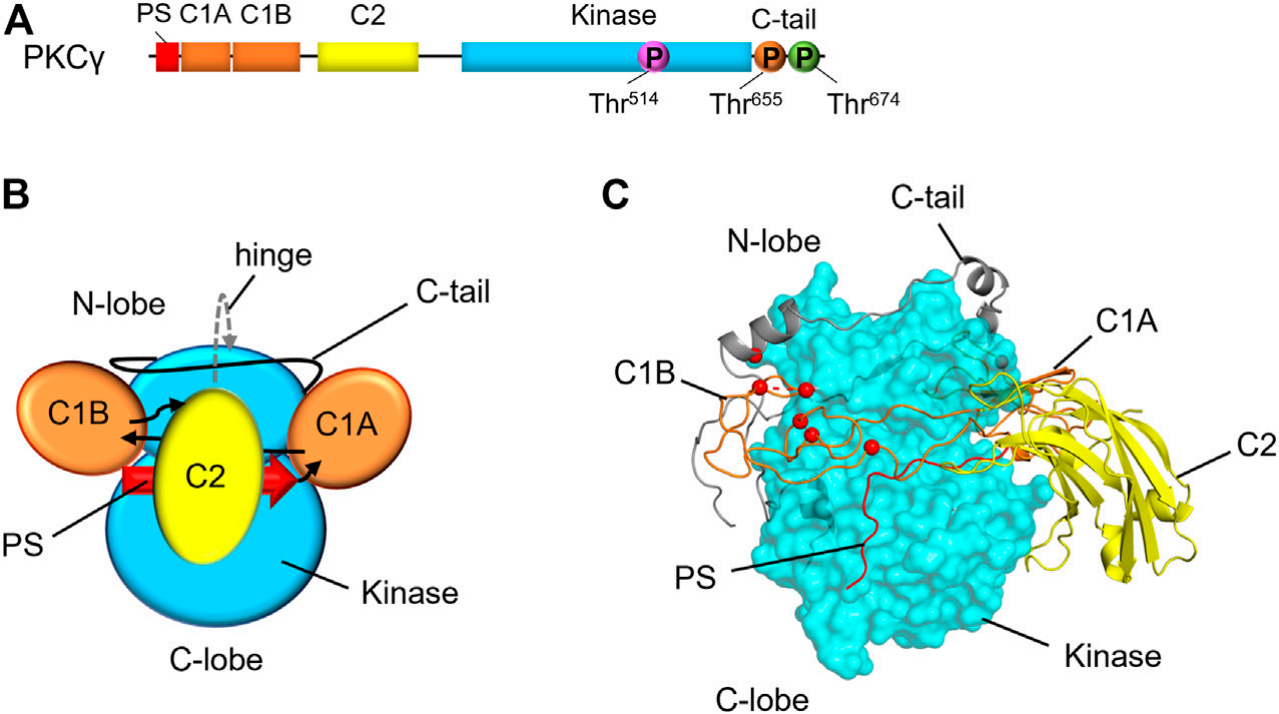
Domain composition and structural model of PKCγ. **(A)** Primary structure of PKCγ, including the pseudosubstrate (PS, red), C1A and C1B (orange), C2 (yellow), kinase (cyan), and C-tail (black line). Circles indicate the priming phosphorylation sites: activation loop (pink), turn motif (orange) and hydrophobic motif (green). This structure is conserved amongst conventional PKC isozymes with a noteworthy difference is a short Pro-rich extension of the C-tail for PKCγ. **(B)** Domain architecture of conventional PKCs with domains labeled. Arrows indicate linker direction. **(C)** Hypothetical model of PKCγ structure based on the previously published model for general architecture of PKC isozymes (Jones et al., 2020; Pilo et al., 2022), showing kinase domain as cyan surface, and the C1 domains and C2 domains in ribbon representation. SCA14 mutations, represented as red spheres, are concentrated in C1B domain or interfaces with the kinase domain.

PKC isozymes share similar domain composition, including a regulatory N-terminal region, a hinge region, and a C-terminal kinase domain (Figure 1A). Contained within the regulatory N-terminal moiety, the pseudosubstrate region binds within the kinase domain active site pocket and prevents signaling in the absence of appropriate second messengers. The regulatory C1 domains bind diacylglycerol (DG) with varying affinity depending on the isozyme class (conventional, novel, or atypical), and they contribute to maintaining PKC in an autoinhibited conformation (Newton, 2018). The C1 domains also serve as docking sites for PHLPP and are required for PHLPP-mediated quality control of PKC (Gao et al., 2008). The C2 domain packs against the kinase domain to keep the pseudosubstrate in the active site pocket until, in conventional PKC isozymes, it binds Ca^2+^ and allows PKC to engage with PIP_2_ at the plasma membrane (Antal et al., 2015a).

Although PKC domain structures have been solved, the full-length structure and the 3D architecture of PKC has yet to be fully elucidated. A partial crystal structure of PKCβII was previously solved, however multiple domains remained unresolved due to inadequate electron density (Leonard et al., 2011). Refining this structure, Leonard and others concluded that conventional and novel PKC isozymes share a common 3D architecture, demonstrating that conserved clamps pack the C1 and C2 domains against the kinase domain (Lučić et al., 2016). In this study, the authors found that mutating certain residues of the PKCβII C2 domain led to faster translocation, suggesting that these residues make up a C2-kinase domain interface (Lučić et al., 2016). A study by Antal et al. mutated lysine residues on the same face of the C2 domain that led to increased translocation of PKCβII (Antal et al., 2015a). Putting the pieces together from these biochemical studies, Kornev and others proposed a conventional and novel PKC structural model (Jones et al., 2020) (Figure 1B). Because the N- and C-termini of each regulatory domain are in close proximity, the authors hypothesized that the regulatory domains would be “plugged in” to the kinase domain to form a common 3D architecture. This hypothetical structure provides a framework upon which other PKC isozymes can be modeled. In the context of PKCγ, for which no structure has yet been solved, this hypothetical structure allows for modeling of disease-associated mutations and predictions for how these mutations would affect PKCγ biochemistry (Figure 1C).

The C-tail of AGC kinases acts to modulate catalysis and to mediate regulatory protein interactions (Kannan et al., 2008).The C-tail wraps around the kinase to structure the enzyme, enables ATP binding, and assists in substrate engagement (Kannan et al., 2008). In PKC isozymes, C-tail phosphorylation at the turn motif and hydrophobic motif is critical for kinase stability (Baffi et al., 2019). PDK-1 docks on the C-tail of PKC to phosphorylate the activation loop (Gao et al., 2001). The C-tail also serves as a docking site for peptidylprolyl cis/trans isomerase, NIMA-interacting 1 (Pin1), which regulates PKC downregulation (Abrahamsen et al., 2012), heat shock protein 90 (Hsp90) and cell division cycle 37, Hsp90 cochaperone (Cdc37), which mediate PKC maturation through the C-tail PxxP motif (Gould et al., 2009), and mTORC2, which phosphorylates PKC at the turn motif and mTOR-interacting motif (TIM) (Cameron et al., 2011; Baffi et al., 2021). In solution, the isolated C-tail is intrinsically disordered, but adopts a helical structure with mixed micelles, as the C-tail tethers PKC to membranes during maturation (Yang and Igumenova, 2013). The C-tail is also one of the most variable regions between PKC isozymes, which is likely critical for determining isoform specificity, given the high sequence similarity in other domains between isoforms (Yang and Igumenova, 2013). PKCγ is particularly interesting in this regard, exhibiting the longest C-terminal tail of the conventional isozymes with an approximately 20 amino acid extension over that of PKCα and βII. The C-tail of PKCγ is particularly proline-rich, with the C-terminal extension additionally containing a PVPVPV repeat. This region has yet to be fully characterized, but this proline-rich region likely mediates protein-protein interactions with PKCγ, such as those involving DGK isozymes (Houssa et al., 1997; Yamaguchi et al., 2006).

### PKCγ in Cancer

In the 1980s, Nishizuka and others discovered that PKC isozymes are the receptor for the tumor-promoting phorbol esters (Castagna et al., 1982), which formed the basis of the dogma that PKC isozymes act as oncogenes. Inhibitors for PKC were developed for treatment of various cancers, yet in clinical trials, they were not only ineffective in treating cancer, but worsened patient outcome. Indeed, a clinical trial meta-analysis for non-small cell lung cancer showed that PKC inhibitors combined with chemotherapy worsened patient outcomes compared with chemotherapy alone (Zhang et al., 2015). A comprehensive study of cancer-associated mutations in every PKC isozyme revealed that PKC mutations in cancer are generally loss-of-function (Antal et al., 2015b). Furthermore, high levels of PKC protein are associated with improved survival in diverse cancers (Tovell and Newton, 2021), reframing PKC as having tumor suppressive properties. Although phorbol esters acutely activate PKC, they lead to the long-term loss of the kinase, so their tumor-promoting properties may arise from their downregulation of PKC (Newton and Brognard, 2017). Thus, restoring PKC function may be a more promising therapeutic avenue for cancer therapy.

Typically, PKCγ is only expressed in neuronal cell types, particularly in the cerebral cortex, hippocampus, and cerebellum (Saito et al., 1988; Saito and Shirai, 2002; Gomis-González et al., 2021). However, evidence for aberrant PKCγ expression has been established in certain cancer types, such as colon cancer and breast cancer (Parsons and Adams, 2008; Garczarczyk et al., 2010; Dowling et al., 2017; Alothaim et al., 2021). Although the mechanism that triggers anomalous PKCγ expression in cancer remains unclear, several studies have addressed the role of PKCγ in these cell types. Specifically, Kiely and others demonstrated that PKCγ knockdown in colon cancer cell lines HT-29 and HCT-116 inhibited cell migration and growth in 2D and 3D (Dowling et al., 2017). However, the HCT-116 cell line contains mutations in PKCγ (Barretina et al., 2012; Nusinow et al., 2020), suggesting that growth inhibition may have arisen from knockdown of a mutated PKCγ. Additionally, PKCγ has been found to be expressed and stabilized in several colon cancer cell lines with the addition of butyrate – a short-chain fatty acid present in the colon at millimolar concentrations (Garczarczyk et al., 2010). Parsons and Adams elucidated a possible mechanism by which aberrantly expressed PKCγ may promote colon cancer cell migration, showing that PKCγ interacted with the tumor-promoting fascin (Parsons and Adams, 2008). On the other hand, in the context of triple negative breast cancer (TNBC), PKCγ has been shown to promote HDAC6 inhibitor-mediated lethality of non-mesenchymal TNBC (Alothaim et al., 2021). Thus, some studies have led to the conclusion that PKC promotes growth, but other factors, like mutations in PKCγ, have not been accounted for.

Mutant PKC has been previously found to have a dominant-negative effect on other PKC isozymes by preventing their processing by phosphorylation, likely because processing requires common titratable elements (Garcia-Paramio et al., 1998). Indeed, many colon cancer cell lines express unphosphorylated PKCγ that is only phosphorylated when butyrate is present, suggesting that mutated PKCγ may act in a dominant-negative manner in these cells (Garczarczyk et al., 2010). Furthermore, short-term treatment with PKCγ C1B domain peptides decreases anchorage-independent growth in the colon cancer cell line COLO205, while increasing expression of other PKC isozymes and p53 (Kawabata et al., 2012). Longer treatment with these peptides decreases PKCα and p53 expression (Kawabata et al., 2012). Thus, mutant PKCγ that is not properly processed and autoinhibited, may lead to global PKC downregulation. Thus, in further studies on the role of PKCγ in cancer, it will be critical to address the effects of PKCγ mutations and how they may be affecting other PKC isozymes.

### PKCγ in Ataxia

Aberrant PKCγ also drives the pathology of a subtype of spinocerebellar ataxia (SCA). SCAs consist of a group of approximately 40 subtypes, all of which are characterized by cerebellar atrophy caused by Purkinje cell (PC) degeneration, resulting in loss of motor coordination and control (Sun et al., 2016). Each SCA subtype is caused by variants in different genes, thus, diagnosis with a specific SCA subtype requires genomic sequencing. Variants in the gene encoding PKCγ (*PRKCG*) are associated with SCA subtype 14 (SCA14) approximately 20 years ago (Yamashita et al., 2000; Chen et al., 2003; Yabe et al., 2003). To date, approximately 50 variants in PKCγ have been identified as causative for SCA14, with most mutations occurring in the C1A and C1B domains (Adachi et al., 2008; Wong et al., 2018; Shirafuji et al., 2019; Schmitz-Hübsch et al., 2021).

The role of aberrant PKCγ in SCA14 has been the subject of much investigation over the past two decades. Early studies established a clear role of PKC activation in PC degeneration. Specifically, studies with organotypic slice cultures from mouse cerebellum showed that phorbol ester treatment leads to PC dendrite degeneration, whereas PKC inhibition leads to an increased dendrite formation and decreased apoptosis (Ghoumari et al., 2002; Schrenk et al., 2002). How PKC activation causes this degeneration remains to be established. However, unbiased network analyses and mechanistic studies provide important clues. One commonality between SCA subtypes may be altered synaptic signaling involving PKCγ, as suggested by network analyses by Verbeek and others (Nibbeling et al., 2017). There is evidence to suggest that this altered signaling may involve diacylglycerol kinase γ (DGKγ). Importantly, PKCγ regulates DGKγ *via* phosphorylation, enabling DGKγ to metabolize DG into phosphatidic acid (Yamaguchi et al., 2006). Specifically, DGKγ knockout mice exhibited PC dendrite degeneration, which was reversed by conventional PKC inhibition (Tsumagari et al., 2020). These mice also exhibited impaired long-term depression (LTD), a critical process in synaptic plasticity. Notably, LTD is known to be induced by PKCα (Leitges et al., 2004), but not PKCγ (Chen et al., 1995). Thus, mutant PKCγ may reduce PKCα function, and therefore LTD induction, *via* decreased cellular DG. Corroborating this, one study found impaired LTD induction and a decrease in depolarization-induced PKCα membrane residence time in PCs expressing a SCA14-associated mutant PKCγ, S119P (Shuvaev et al., 2011). Although PKCγ may drive SCA14 by other mechanisms, these studies suggest that enhanced PKCγ activity drives SCA14 in a DGK-dependent-manner.

How the diverse SCA14 mutations alter PKC function has also been the subject of numerous studies culminating in a recent comprehensive analysis of approximately 50 variants (Pilo et al., 2022). This study concluded that ataxia-associated PKCγ mutations enhance basal activity, as mutations in each domain of PKCγ had impaired autoinhibition (Pilo et al., 2022). Although defects in autoinhibition generally lead to PKC degradation, this study demonstrated that C1 domain mutations protect PKCγ from phorbol ester-induced downregulation. Additionally, the degree of impaired autoinhibition correlated inversely with average age of disease onset in patients, supporting a role for disrupted PKCγ autoinhibition in SCA14 (Pilo et al., 2022). A previous study of SCA14-associated PKCγ mutations demonstrated that SCA14-associated mutations unmasked the C1 domains to increase PKCγ membrane translocation (Verbeek et al., 2005, 2008); using a genetically-encoded PKC activity reporter (Violin et al., 2003), the authors showed reduced amplitude of agonist-evoked activation of PKCγ SCA14 mutations leading the authors to suggest that the SCA14 mutations had impaired activity. However, basal activity was not addressed in this study, and later analysis showed that basal activity, rather than agonist-indued activity drives SCA14 (Pilo et al., 2022). Another study expressing various SCA14-associated mutants in PCs *in vitro* demonstrated no effect of the mutants on dendritic development, concluding that enhanced activity of PKCγ was not required for SCA14 pathogenesis (Shimobayashi and Kapfhammer, 2017). Studies on G360S, a variant occurring in the kinase domain of PKCγ, have also produced conflicting reports. Whereas Adachi et al. found that this mutant is not activated by Ca^2+^ (Adachi et al., 2008), Ueno and others demonstrated that G360S was more basally active and had higher agonist-stimulated activity compared to wild-type PKCγ (Asai et al., 2009). A mutant that generates an early stop in the C1A domain of PKCγ (R76X) leads to elimination of PKCγ activity, however, this fragment may activate other PKC isozymes *via* RACKs (Shirafuji et al., 2019). Aggregation of PKCγ mutants in SCA14 has also been a focus within the field. Specifically, overexpression studies of wild-type and mutant PKCγ have been shown to form toxic fibrils and aggregates, the occurrence of which was reduced by stimulation of heat shock proteins (Takahashi et al., 2015; Nakazono et al., 2018). Aggregates of endogenous mutant PKCγ have also been detected in SCA14 patient-derived iPSCs (Seki et al., 2009; Wong et al., 2018). However, the interplay between altered PKCγ activity and aggregation has yet to be elucidated. Thus, whereas the aforementioned previous studies have proposed a variety of mechanisms that may be involved in the cerebellar degeneration that is characteristic of ataxia, the previously established correlation between enhanced basal activity of PKCγ variants with age of disease onset support a model in which increased PKCγ signaling in the absence of second messengers likely drives SCA14 (Pilo et al., 2022).

Mouse models of ataxia generated by Kapfhammer and others have demonstrated that PKCγ mutations drive SCA14 pathogenesis (Ji et al., 2014; Trzesniewski et al., 2019; Shimobayashi and Kapfhammer, 2021). The first of these mouse models was the S361G transgenic mouse, which was shown to exhibit an ataxic phenotype and reduction of PC surface area (Ji et al., 2014). They also generated a transgenic mouse expressing a pseudosubstrate mutant PKCγ, A24E, which caused an ataxic phenotype and weakened PC development (Shimobayashi and Kapfhammer, 2021). Mutations in the pseudosubstrate generally decrease its affinity for the active site, thus destabilizing PKC (Baffi et al., 2019). Although the A24E mutation reduced PKCγ stability, the basal activity of the A24E mutant PKCγ increased cerebellar substrate phosphorylation and was sufficient to drive an ataxic phenotype. Thus, these mouse models have supported the idea that increased PKCγ activity may be a main driver of SCA14 pathology.

## Conclusion

**FIGURE 2:**
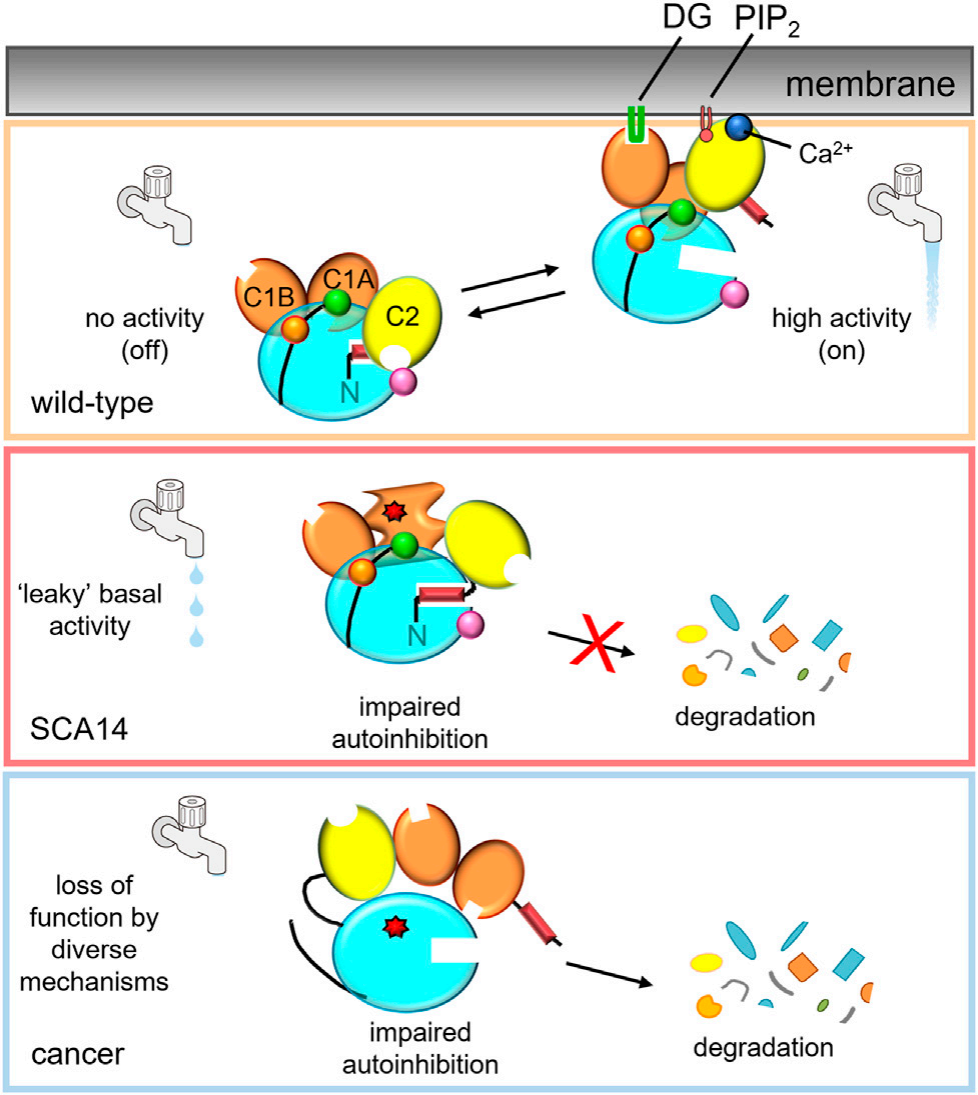
PKCγ mutations in disease lead to differing effects on kinase activity.Top: In the absence of second messengers, wild-type PKCγ adopts an autoinhibited conformation, in which no signaling occurs (water faucet is “off”). In the presence of Ca^2+^ and DG, wild-type PKCγ adopts an open conformation and is activated (water faucet is “on).Middle: Mutations in SCA14 lead to impaired autoinhibition of PKCγ resulting in “leaky activity”; mutations in the C1 domains protect PKC from down regulation, evading quality control degradation of the impaired PKC. This species can be further activated by second messenger binding for some, but not all, SCA14 mutations (not shown). Bottom: Mutations in cancer lead to loss of PKC function by diverse mechanisms. One common mechanism is by impairing autoinhibition, resulting in the dephosphorylation and degradation of PKC. Mutant PKCγ can also act in a dominant negative manner to suppress signaling by other PKC isozymes.

PKCγ is best understood in the context of the neurodegenerative disorder, SCA14, however, many gaps in our knowledge of this PKC isozyme remain. Despite belonging to the generally well-studied group of conventional PKC isozymes, limited attention has been given to the aberrant expression of PKCγ in colon cancer, in particular. Furthermore, whereas several studies have reported that knockdown of this enzyme in cancer cells inhibited proliferation and foci formation in 3D, many of these cancer cell lines have somatic mutations in PKCγ that were not addressed. Given that PKC mutations in cancer generally are not only loss of function, but also dominant-negative, gaining a better understanding of cancer-associated PKCγ mutations will be critically important to applying therapies that will produce beneficial outcomes in cancer patients harboring these mutations (Figure 2). Gaps also exist in our understanding of the role of PKCγ in SCA14. Although mechanistic studies converge on enhanced PKCγ basal activity driving SCA14 pathogenesis, how this leaky activity leads to PC degeneration remains to be established (Figure 2). Elucidation of the structure for PKCγ will also greatly advance our understanding of this isozyme. Although the theoretical models and partial crystal structures that have been generated are currently helpful tools in predicting mutational effects, these are based on the better-characterized PKCβII. Despite many PKC isozymes sharing a common 3D architecture, the subtle primary sequence differences as well as the highly variable C-tail likely alter factors such as interdomain interactions, subcellular localization, and substrate preferences. The impact disease-specific mutations have on these factors for PKCγ, specifically, will be difficult to fully grasp until a structure is fully solved.
